# Male and Couple Fertility Impairment due to HPV-DNA Sperm Infection: Update on Molecular Mechanism and Clinical Impact—Systematic Review

**DOI:** 10.1155/2014/230263

**Published:** 2014-03-25

**Authors:** Salvatore Gizzo, Bruno Ferrari, Marco Noventa, Emanuele Ferrari, Tito Silvio Patrelli, Michele Gangemi, Giovanni Battista Nardelli

**Affiliations:** ^1^Department of Woman and Child Health, University of Padua, 35128 Padua, Italy; ^2^Department of Surgical Sciences, University of Parma, 43100 Parma, Italy; ^3^Dipartimento di Salute della Donna e del Bambino, U.O.C. di Ginecologia e Ostetricia, Via Giustiniani 3, 35128 Padova, Italy

## Abstract

Recent evidences identify Human Papillomavirus (HPV) sperm infection as a possible cause of male and couple infertility. It acts through different mechanisms at various steps of human conception and early gestational development. We performed a systematic review to assess the role of HPV semen infection on male and couple infertility. Analysis of available and eligible data does not permit us to fund clear evidences about clinical impact of HPV infection on fertility, although sperm parameters impairment is the most widely recognized effect. Regarding biomolecular implications, the available data are often conflicting. More studies are required to define the role of HPV sperm infection in clinical practice. The great majority of evidences are obtained by *in vitro* studies and this fact represents a limitation for the clinical management of HPVDNA sperm infection. Understanding the biological significance of HPV-DNA semen infection could permit us to explain most of the idiopathic male and couple infertility, leading to a better management of infertile men and a better timing for sperm banking storage before ART cycles.

## 1. Introduction

Human Papillomavirus (HPV) infection is one of the main sexually transmitted diseases worldwide [1]. The most important clinical consequence of HPV infection is cervical cancer which remains one of the leading causes of cancer-related mortality in young and older women [2–5].

HPV infection is also related to anogenital warts and different neoplasia, such as vaginal, vulvar, penile, anal, oral cavity, head, and neck cancers [1, 4, 6]. Considering male and female, the overall prevalence of HPV infection is about 40% of population, with differences based on the HPV type and the anatomical site of infection [7].

Great progresses have been reached in understanding the pathologic mechanisms of HPV infection. Both effective screening programs (pap smears, HPV-DNA testing) and interventions (HPV vaccination) have been developed in order to reduce HPV related disease in women [8–12].

Nevertheless few data are available on male infection [1, 13–15].

Recent findings underlined the role of HPV semen infection in male and couple infertility, focusing mainly on its high prevalence among 18–40 years old men [16, 17].

The exact localization of HPV in the spermatozoa is not well defined, even if recent studies demonstrated that the virus binds two distinct sites along the equator of the spermatozoa's head [18, 19].

Many Authors hypothesized that HPV can modify spermatic parameters causing sperm motility reduction, seminal pH alterations, and spermatozoa DNA fragmentation [18, 20–22].

Although* in vitro* studies demonstrated that spermatozoa can carry HPV-DNA and transfer it to oocytes, it is still not clear if* in vivo *the HPV-infected sperm is able to fertilize oocyte and to transfer the viral genome [17, 23–25]. These uncertainties extend to the following steps of conception because it is not clear if the infected oocytes are able to generate normal embryo and if the infection itself could interfere with implantation and subsequent pregnancy development [17].

The aim of this review is to investigate the implications of HPV sperm infection on male and couple infertility, analyzing the clinical impact on early pregnancy development and pregnancy loss, the paradigmatic spermatic alterations, the sperm immunological modifications, and the spermatozoa HPV-related molecular changes. Finally we analysed the effect of HPV-infected sperm on fertilized oocyte, blastocyst implantation, and pregnancy development. We will also discuss the available diagnostic and therapeutic strategies, in terms of feasibility and clinical efficacy trying to make some considerations on sperm banking before ART cycles.

## 2. Data Sources

A Literature analysis was performed on the electronic databases Medline, Embase, ScienceDirect and the Cochrane Library, considering papers published in the time interval from 1994 to 2013.

We looked for randomized trials, observational and retrospective studies, original works, and review articles having topics as the relation between male HPV sperm infection, seminal modifications, effects on fertilized oocytes, association to apoptosis, early miscarriages, and implications on male and couple reproductive outcomes.

Key-terms included “HPV sperm infection,” “male infertility and HPV,” “sperm parameters and HPV,” “HPV infected sperm and fertilization,” “HPV and fertility outcome,” and “HPV and blastocyst apoptosis.” An accurate analysis of the references of the main works was successively performed.

We considered data from eligible studies separately, according to different topics, “clinical impact of HPV infection and fertility outcomes,” “HPV-related spermatic modifications and their impact on fertility,” and “ability of infected semen to vehicle exogenous HPV-DNA and its impact upon ongoing pregnancy.”

## 3. Methods

We evaluated the clinical significance of genital HPV-DNA presence in male, female, and couple in relation to placental infection (both at term and preterm) and spontaneous miscarriage (Table 1).

The seminal parameters were defined according to WHO laboratory manual for examination and processing of human semen [5] and Hamilton Thorn motility analyser [42].

We analysed both clinical and experimental works which focused on the ability of human sperm to vehicle HPV-DNA infection into oocytes and subsequent implications (Table 2).

HPV-DNA detection in various sample tissues was based on polymerase chain reaction (PCR) and fluorescence in situ hybridization (FISH) techniques.

The sperm washing techniques reported in the studies were two-layer isolate colloid wash, test-yolk buffer procedures, swim-up procedure, modified swim up with enzymatic treatment (Heparinase-III), and discontinuous Ficoll gradients. We included also studies which used DNA disc chip assay, Comet assay, and Cell Death Detection ELISA in order to detect HPV-related cells apoptosis.

Finally the hamster egg-human sperm penetration test (HEPT) has been used in some works in order to assess the ability of HPV-infected sperm to fertilize and to transfer viral genome into the oocytes.

## 4. Results

According to our selection criteria, only 23 works had eligible results for the aim of the review.

### 4.1. Clinical Impact of HPV Infection and Fertility Outcomes

Only 5 papers focused on this topic (Table 1). Hermonat et al. in 1997 firstly performed a study evaluating the presence of HPV-DNA in 25 spontaneous early miscarriages versus 15 voluntary abortions. 15 of the 25 spontaneous samples (60%) were found to be positive for HPV E6/E7 sequences versus only 3 of the 15 elective samples (20%). Semiquantitative analysis showed that the HPV detection was six-fold higher in the spontaneous abortions compared to elective ones (*P* < 0.01) [26].

Matovina et al. analyzed the products of conception from early miscarriage detecting a HPV 16–18 incidence of 7.4% [27].

Dana et al. focused on the exposure to HPV type 6/11/16/18 during pregnancy and found a spontaneous abortion rate of 6.9%, a prevalence of major birth defects of 2.2% and fetal death rate of 1.5% [28].

In 2010 Skoczynski et al. compared the prevalence of HPV infection in placentas from term deliveries and spontaneous abortions. The comparison between the two groups showed no differences in terms of HPV-DNA detection since it was found in 24.4% of placentas at term (12.8% of HPV 16/18 types) and in 17.7% of miscarriages (11.8% of HPV 16/18) [*p* : *n*.*s*.] [29].

Perino et al. in a cohort of 199 couples undergoing ARTs reported a prevalence of 9.5% (19/199) for HPV male infection, 17.5% (35/199) for female infection, and 4.5% (9/199) for both partners infection. After ARTs, miscarriage rate had considerably higher results in couples where a single or both partners were HPV carriers compared to noninfected couples. The comparison between the two groups resulted in 66.7% versus 15% in case of male infection (*P* < 0.01), 40% versus 13.7% in case of female infection, and 100% versus 15.9% in case of both partners infections (*P* < 0.001) [30].

The main limitations of all these studies are certainly related to the small sample size. Moreover, all the considered studies were retrospective or cross-sectional since the only perspective one was performed by Perino et al. [30].

### 4.2. HPV-Related Spermatic Modifications and Their Impact on Fertility

Only 9 eligible studies focused on this topic (Tables 2 and 3).

Both Brossfield et al. and Connelly et al. analysed the spermatic effects of HPV infection, showing that sperm cells transfected with exogenous HPV-DNA had higher percentages of total motility and progression compared to the untreated controls (*P* < 0.05) [21, 31].

On the contrary, Lee et al. demonstrated that sperm motility was reduced after HPV E6-E7 fragments expression. The percentages of progressive motility were lower in sperm exposed to all the HPV-DNA genotypes (except for the 33); the amplitude of lateral head displacement was decreased after exposure to (high-risk) hrHPV-DNA type 16 and all (low-risk) lrHPV-DNA (*P* < 0.05) [22].

In 2004 Rintala et al. analysed 65 sperm donors, 15.5% results were positive for hrHPV-DNA and 17% positive for lrHPV-DNA. They reported no HPV-related effects on sperm motility and concentration, except for pH changes [20].

Foresta et al. evaluated 200 male sperm donors observing that sperm parameters results were similar between the two groups except for the sperm motility which was significantly reduced in HPV-DNA infected men (*P* < 0.05) [18].

In the same year, Foresta et al. showed that HPV-DNA was frequently detected in exfoliated epithelial cells of lower genital tract [77.8–100%], while spermatozoa infection was detected in 72% of infertile men. Despite the mean sperm motility results significantly reduced in all infected men compared to noninfected ones (*P* < 0.05), all the remaining parameters were similar between infected or noninfected patients. A reduction of sperm concentration was registered in case of infertility, independently from HPV-DNA infection (*P* < 0.05) [32].

Considering sperm-washing procedure (in order to remove HPV-DNA from the sperm surface) Brossfield et al. showed that all the applied techniques did not positively influence the spermatic HPV-related motility and that, between them, the centrifuge wash technique results were the best ones [31].

On the other hand Foresta et al. observed that only Ficoll and swim-up procedure were useful to reduce the infection in 30% and 26% of sample, respectively (*P* < 0.01) [33].

Garolla et al. demonstrated the negative effects of HPV infection reporting that only progressive sperm motility was significantly reduced in infected semen samples (*P* < 0.05). Authors reported that direct swim-up reduces up to 24% the HPV sperm infection, while modified swim-up is able to remove completely HPV-DNA from spermatozoa (*P* < 0.01). Anyway, Authors underlined how modified swim-up results were responsible for a slight decrease of sperm motility, viability, and DNA integrity [17]. The same author in 2013, using the sperm-Mar test to detect antisperm antibodies (ASA), demonstrated that infertile men showed more frequently ASA than fertile ones (*P* < 0.01). The evidences that men with HPV-DNA infection associated to positive sperm-Mar test are affected by 24 months lower spermatozoa motility than negative ones lead the authors to propose the positive sperm-Mar test as predictor tool for future progressive spermatozoa' motility [34].

### 4.3. Infected Semen Ability to Transmit Exogenous HPV-DNA and Its Impact upon ongoing Pregnancy

Only 11 studies analysed sperm ability to carry exogenous HPV-DNA into oocytes and viral genome into the blastocysts and its impact in terms of fertility, implantation, and embryonic effects (Table 4).

Even if Chan et al. in 1994 showed that human sperm could be infected by HPV-DNA, Lai et al. in 1996 first assumed that spermatozoa could act like a vector for HPV transmission to sexual partners and to foetus through fertilized eggs [35, 43].

Later, both Connelly et al. and Lee et al. demonstrated increased apoptotic phenomena in sperm cells exposed to E6/E7 genes of HPV-DNA types 16 and 18 (*P* < 0.05) [21, 22].

Chan et al. demonstrated in a mouse experimental model the ability of HPV-DNA infected sperm to transmit its genome to blastocysts [37]. Cabrera et al. demonstrated the presence of HPV-DNA in both the inner cell mass and trophoblastic cells of murine infected blastocysts [23].

Foresta et al., using the hamster egg-human sperm penetration test (HEPT), demonstrated the sperm ability to transfer both the capsid protein L1 and E6/E7 viral genes to oocytes with a subsequent gene expression by transfected blastocysts [25].

Calinisan et al., transfecting blastocysts with the E6-E7 region of types 16, 18, 31, and 33, detected the presence of DNA fragmentation only in the subgroup of blastocysts infected by HPV-DNA type 16 (*P* < 0.05). The ability of HPV-DNA type 16 to induce DNA fragmentation and subsequent trophoblastic death was also confirmed by You et al. in 2002 [38].

Hennemberg et al. confirmed the direct HPV16 inhibitory effect on blastocysts growth only at the two-cell embryo stages but not on the 4–8 cells ones [40].

Gomez et al. showed that the apoptosis rate in transfected trophoblastic cells was 3-fold (2.4–3.7) and 5.8-fold (5.6–5.9) greater at 3 and 12 days, respectively, if compared to negative controls (*P* < 0.01). Simultaneously, authors reported that the invasion ability of transfected trophoblast progressively decreased from day 3 to day 15 (25.2–57.6% lower than negative controls) (*P* < 0.001) [41].

The limitations of all these studies were certainly related to the artificial conditions linked to the* in vitro* experiments and murine models which may not reflect the real* in vivo* human situations. All these data should be validated by large* in vivo* perspective studies but unfortunately the literature lacks information about this field because of ethical policies concerning human reproduction experiments.

## 5. Discussion

It is widely proven that sexually transmitted infections (STD) represent a possible cause of male infertility since they can induce urethral stickiness, epididymis inflammation, and orchitis up to testicular failure [44]. The impairment of sperm motility and DNA integrity through an autoimmune mechanism could result in obstetric complications such as early miscarriages and preterm deliveries [27, 45].

The high prevalence of male HPV detection raised strong interest for its possible consequences on male fertility. HPV infection natural history in men is still mostly unknown. Recent findings suggest that the incidence of genital HPV infection is 38.4 per 1000 male/months (95% CI 34.3–43.0). The mean duration of male infection results of 7–52 months are (6.80–8.61) for any HPV type and of 12–19 months (7.16–18.17) are for hrHPV 16 [46].

### 5.1. Clinical Impact of HPV Infection and Fertility Outcomes

All* in vitro* studies showed a negative influence of HPV upon several aspects of male fertility. However, only few studies investigated the* in vivo* effects of HPV on human reproduction phases. In 1997 Hermonat et al. reported the correlation between HPV infection and spontaneous miscarriages in first trimester of pregnancy. The higher percentage of HPV-DNA detection, if compared to voluntary abortions, paved the way to consider HPV as one of the possible etiologic agents responsible for early pregnancy loss [26]. This hypothesis was not confirmed by Matovina et al. who found HPV-DNA in only 7.4% of spontaneous miscarriage specimens. Anyway the authors did not exclude the possibility of HPV-DNA trans-placental transfer [27].

Nowadays, in spontaneous conception, the rate of early miscarriages and major birth defects does not seem greater in HPV-exposed couples than in unexposed ones [28, 29]. However, considering* in vitro* fertilization techniques, this aspect seems to be of crucial importance. The first prospective study on this topic reported a significant pregnancy loss increase in couples undergone ARTs and male partner with semen HPV infection (miscarriage rate of 100% when both partners results are infected) [30].

So we can conclude that the role of HPV infection in spontaneous abortion is still not clear and its association with adverse pregnancy outcomes is not certainly demonstrated. Further perspective longitudinal studies are necessary to better understand the possible roles of HPV infection in early miscarriage and in other adverse pregnancy outcomes.

However, if the evidences of Perino et al. will be confirmed, a major care on HPV status of couples attempting ART procedures should be necessary. Thus, HPV male vaccination could represent a possible strategy for male fertility preservation and for ART success rate improvement.

### 5.2. HPV-Related Spermatic Modifications and Their Impact on Fertility

The molecular mechanisms by which HPV could impair sperm quality and fertilized oocytes development has been neither completely demonstrated nor clarified.

From our analysis, HPV presence in spermatozoa may be associated with an impairment of sperm parameters. Lai et al. firstly demonstrated that HPV presence in sperm could affect spermatozoa's motility (lower velocity, straight-line velocity, and mean amplitude of lateral head displacement) although it seems to be not statistically significant. A possible association between HPV semen infection and asthenozoospermia has also been described [36]. These findings seem to be partially confirmed even by following studies. Previous works showed that prewashed sperm specimens transfected with L1 HPV-DNA fragments had an increased motility and progression but they did not elucidate the possible mechanisms [21, 31]. On the other hand, Lee et al. described a possible association between HPV-DNA presence, sperm motility reduction, and total progression after 24 hours of incubation [22].

The slight motility increase described by Connelly et al. in HPV exposed spermatozoa might be explained considering that the observation was performed after two hours of incubation. This fact suggests that HPV-DNA requires a suitable interval time to determine molecular changes on sperm motility apparatus. The authors reported a mean reduction of lateral head amplitude even if the virus seems not able to decrease oocytes sperm fertilization ability [21].

Rintala et al. in HPV positive semen detected only a pH change without any impairment of other parameters [20].

For many years semen pH impairment was considered the most important mechanism explaining fertility decrease due to asymptomatic genital infections, especially the bacterial ones.

In our opinion pH impairment represents only one of the several factors that may influence male fertility, since the aetiological mechanism could involve many other factors such as mean sperm motility, presence of ASA on the spermatozoa surface, and qualitative semen parameters impairment till asthenozoospermia [18, 32–34].

Since several cases of reported idiopathic asthenozoospermia do not present any known risk factor except for the positivity to HPV-DNA, semen washing procedures could represent a way to improve spermatozoa quality before ART procedures [18, 32, 34].

Starting from these considerations, many studies proposed different semen washing methods to eliminate HPV-DNA sperm infection, but all proposed techniques failed in the scope, except for the modified swim-up technique. This last one contemplates enzymatic treatment (Heparinase-III), apparently able to completely remove HPV-DNA from sperm cells although with a deterioration in semen quality.

Nowadays all the available data suggest that the classic procedures cannot eliminate HPV sperm infection and that HPV-DNA semen screening can be considered only as an epidemiological investigation helping to define the best timing (regression of semen infection) to start ART cycles.

It is well demonstrated both in men and women that HPV infection is generally transient and only few patients are subject to persistent infection. Our suggestion to test the sperm for HPV before sperm banking and ART cycles could represent the last option for the clinicians to try to improve the semen quality both for fresh use and for frozen preservation. Certainly, the vaccine option could have useful results for both male and female patients presenting poor reproductive outcomes till the couple infertility [47].

### 5.3. Ability of Infected Semen to Vehicle Exogenous HPV-DNA and Its Impact on Pregnancy Evolution

The HPV-DNA presence in sperm and the related modification induced by the infection seem to play a role in physiopathology of unexplained male infertility. However,* in vitro* evidences showed that HPV infected spermatozoa maintains the ability to fertilize oocytes and to express viral genome in the product of conception. This suspect seems to be confirmed by a higher rate of* in vitro* blastocysts and trophoblastic HPV-related apoptosis probably responsible of* in vivo* fertility rate reduction of infected couples.

Evidences reported by Chan et al. [35], Lai et al. [43], Pérez-Andino et al. [19], and Foresta et al. [25] clarified many aspects of the overall fertility reduction in couple with male HPV infection. The HPV ability to bind the spermatozoa in two distinct sites of the equatorial region through sydecan-1 (a proteoglycan expressed almost exclusively in the equatorial region of sperm head) can explain the sperm ability to carry viral genome into fertilized oocytes and blastocysts [19, 21, 22, 25].

In both experimental murine and* in vivo* human models it was found that HPV genomes are expressed in fertilized oocytes, blastocysts, and trophoblastic cells [23, 25, 37]. The viral genome could induce cellular changes such as inhibition of zygote growth, decrease in blastocyst formation, inhibition of blastocyst hatching process, and DNA fragmentation and apoptosis, and thus results are often lethal for early embryo development [38–40].

*In vitro* experiments showed that HPV transfected blastocysts and trophoblastic cells were affected by a reduction in decidua invasion capacity, potentially responsible for a failure of maternal uterine wall invasion by the extravillous trophoblastic cells, subsequent placental dysfunction, and adverse pregnancy outcomes (i.e., early miscarriage) [41].

Nowadays both the exact mechanism and timing through which the HPV infection modifies trophoblastic genes expression and increases cell death have not yet been understood. All the considered studies have important limits which should not be underestimated, linked to the* in vitro* artificial and experimental conditions. Further validation in* in vivo *studies could be therefore needed.

### 5.4. New Insight: HPV Male Vaccination and Sperm Effects

The actual high prevalence of HPV sperm infection represents a large scale problem for the sperm donors banks [48, 49].

HPV screening for all semen samples before sperm banking should be considered as a real option of semen storage for future ART cycles.

The rationale is that, when possible, the semen banking should be postponed until HPV infection resolution. The main limitations in this field are linked to the poor knowledge of HPV male infection natural history and of the most adequate interval time to postpone the storage.

Moreover, neither evidences are available about long term effects of previous HPV sperm infection nor which parameters could recover after the infection-linked damages.

In absence of effective and safety sperm washing procedures able to eliminate the infection, HPV male vaccination should be considered as a possible strategy for the prevention of HPV semen impairment and for the improvement of couple fertility outcomes [4, 30, 46, 48, 50, 51].

Male vaccination could represent also a reliable option for couples undergoing ART cycles because fertility of female partner results yet partially compromised: this could solve the problem linked to sperm banking, avoiding the potential HPV negative effect on sperm quality [52].

The biggest concern of Public Health Programs and clinical practitioners about the cost-effectiveness of HPV male vaccination is linked to the several biases affecting available data. For example, a recent study by Kim and Goldie concluded that the HPV vaccination of all 12-year-old boys would not be cost effective [53]. All readers should realize that this conclusion was obtained despite strong bias: oncologic safety and efficacy in men were not included since it considered only women outcomes; only heterosexual couples were considered without including outcomes in homosexual men; it did not evaluate data about incidence, mortality, and quality of life linked to cancers different from cervix and, finally, it did not contemplate the possible role of HPV male infection on fertility.

## 6. Conclusions

Most of analysed data suggested that the HPV sperm infection could be responsible for a decreased fertility rate through different mechanisms acting at various steps of the human embryo development.

The still debated clinical features related to HPV-DNA sperm infection are the increased risk of early miscarriage and the higher incidence of unexplained male infertility (related to sperm parameters impairment). HPV-DNA sperm test should be realized in semen donors and before ART cycles, despite the limitations linked to* in vitro* studies evidences. Improvement in the knowledge of HPV-DNA sperm infection mechanisms, timing, and link to fertility impairment could explain most of the actual “idiopathic” male and couple infertility.

The cost-effectiveness analysis related to fertility improvement in HPV vaccinated male requires further evaluation in the next future.

The implications related to possible achievement of herd immunity after a mass population vaccination program could overcome the existing doubts, explaining and resolving the unclear aspects in this field.

## Figures and Tables

**Table 1:** Data about studies analyzing the clinical impact of HPV infection on fertility outcomes.

Authors (Year)	Number of samples	Spontaneous aborted products: HPV+/HPV−	Electively aborted products: HPV+/HPV−	Incidence of miscarriages in pregnant exposure to HPV 6/11/16/18	Samples of placentas at term: HPV+/HPV−	Incidence of miscarriages in couple with male partner: HPV+/HPV−	Incidence of miscarriages in couple with female partner: HPV+/HPV−	Incidence of miscarriages in couple with both partner: HPV+/HPV−
Hermonat et al. (1997) [26]	40	60%/40%	20%/80%					
Matovina et al. (2004) [27]	108	7.4%/92.6%						
Dana et al. (2009) [28]	517			6.9%				
Skoczynski et al. (2011) [29]	129	17.7%/82.3%^+^			24.4%/75.6%^+^			
Perino et al. (2011) [30]	199 (couple)					66.7%/15%*	40%/13%	100%/15.9%°

^+^ *P* < 0.05; *P < 0.01; °*P* < 0.00.

**Table 2:** Data about **s**tudies analyzing HPV-related spermatic modifications and their impact on fertility according to Hamilton Thorn Motility Analyzer (Data are expressed as value ± SD).

Authors (year)	Type of study	Patients	Total motility (%)	Progressive motility %	Amplitude lateral head (*μ*m)	Percentage hyperactive (%)	Straight-line velocity (mm/sec)	Curvilinear velocity (m/s)	Average path velocity (mm/sec)	Linearity (%)
Brossfield et al. (1999) [31]	Case control study	Sperm exposed to L1 HPV DNA *n* = 523	51.5 ± 0.15*	15.5 ± 0.11*	1.9 ± 0	0.5 ± 0.02	23.5 ± 0.02	43.5 ± 0.02	34.0 ± 0.04	57.0 ± 0.04
		Control sperm washed *n* = 498	75.0 ± 0.45*	19.0 ± 0.04*	3.1 ± 0	4.0 ± 0.09	24.5 ± 0.07	57.5 ± 0.02	41.0 ± 0	44.5 ± 0.11
		Transfected centrifuge-washed *n* = 114	90.0 ± 0	38.5 ± 0.61	4.1 ± 0.03	8.0 ± 0.38	31.5 ± 0.2	73.5 ± 0.05	47.5 ± 0.05	44.5 ± 0.14
		Transfected Isolate-washed *n* = 102	93.0 ± 0.10	33.5 ± 0.05	3.6 ± 0.12	1.0 ± 0.10	26.0 ± 0.10	56.5 ± 0.25	37.5 ± 0.25	47.0 ± 0
		Transfected, TYB-washed *n* = 103	94.0 ± 0.40	37.0 ± 0.70	3.7 ± 0.01	4.0 ± 0	26.0 ± 0.10	58.5 ± 0.05	36.0 ± 0.20	46.5 ± 0.35

Connelly et al. (2001) [21]	Case control study	Patients HPV-DNA 16 size = 98	48.0 ± 0.2*	5.5 ± 0.2*	1.8 ± 0*	1.0 ± 0.1		38.5 ± 0.3	28.0 ± 0.1	56.0 ± 0.2*
		Patients HPV-DNA 18 size = 80	47.5 ± 0.1*	11.0 ± 0.2*	2.7 ± 0*	1.0 ± 0.1		55.5 ± 0.7	36.0 ± 0.5	47.5 ± 0.2*
		Patients HPV-DNA 6b/11 size = 157	36.5 ± 0.1*	6.5 ± 0.1*	1.8 ± 0*	0 ± 0		31.5 ± 0.1	23.0 ± 0	57.0 ± 0.2*
		Patients HPV-DNA 31 size = 162	55.0 ± 0.5*	14.5 ± 0.1*	2.8 ± 0.1*	2.0 ± 0		45.5 ± 0.3	30.5 ± 0.1	52.5 ± 0.5*
		Patients HPV-DNA 33 size = 103	48.5 ± 0.7*	13.0 ± 0.3*	2.7 ± 0*	0 ± 0		42.5 ± 0.4	28.5 ± 0.3	52.5 ± 0.2*

Lee et al. (2002) [22]	Case control study	control subjects size = 449	74.0 ± 0.6*	17.3 ± 0.2*	3.0 ± 0.0*	2.3 ± 0.1		58.4 ± 0.2		
		Patients HPV-DNA 16 size = 433	38.4 ± 1.1*	6.0 ± 0.2*	1.3 ± 0.1*	1.4 ± 0.1		40.1 ± 0.7		
		Patients HPV-DNA 18 size = 436	56.1 ± 0.5*	9.1 ± 0.2*	2.9 ± 0.0*	2.3 ± 0.1		53.0 ± 0.2		
		Patients HPV-DNA 6/11 size = 451	47.1 ± 0.5*	7.0 ± 0.2*	2.1 ± 0.0*	3.3 ± 0.1		51.4 ± 0.4		
		Patients HPV-DNA 31 size = 434	53.4 ± 0.6*	8.6 ± 0.1*	2.7 ± 0.0*	2.4 ± 0.1		52.0 ± 0.4		
		Patients HPV-DNA 33 size = 424	46.0 ± 1.3*	10.8 ± 0.4*	3.0 ± 0.0*	2.9 ± 0.1		47.3 ± 1.3		
		controls DQA1 size = 437	47.0 ± 0.6*	6.5 ± 0.1*	2.7 ± 0.0*	4.3 ± 0.3		49.6 ± 1.0		

*P < 0.05.

**Table 3:** Data about studies analyzing HPV-related spermatic modifications and their impact on fertility according to WHO laboratory manual for the examination and processing of human semen (Data are expressed as value ± SD).

Authors (year)	Study type	Patients	Sperm concentration (10^6^/mL)	Semen volume (mL)	Total sperm count (10^6^)	pH	Progressive motility %	Normal morphology %	Viability %
Rintala et al. (2004) [20]	Case control	High-risk HPV DNA (+) *n* = 10		3.07		7.37	54.2		65.2
		High-risk HPV DNA (−) *n* = 55		4.03		7.51	56.5		69.6

Foresta et al. (2010a) [18]	Cross-sectional clinical	Sexually active subjects HPV (+) *n* = 10	57.5 ± 30.4	2.9 ± 1.6	174.3 ± 115.8	7.7 ± 0.3	37.7 ± 16.8*	31.5 ± 8	83.5 ± 7.9
		Sexually active subjects HPV (−) *n* = 90	60.2 ± 31.0	2.4 ± 1.6	175.8 ± 154.5	7.6 ± 0.2	53.7 ± 18.2*	33.1 ± 11.1	84.6 ± 8.6
		Virgin subjects *n* = 100	58.3 ± 29.1	2.7 ± 1.5	174.5 ± 164.7	7.6 ± 0.3	53.7 ± 19.0*	32.8 ± 10.6	83.6 ± 7.6

Foresta et al. (2010b) [32]	Cross-sectional clinical	Patients with genital warts HPV (+) *n* = 14	53.5 ± 30.0*	2.6 ± 1.7	167.6 ± 111.7	7.7 ± 0.2	36.2 ± 18.7*	32.6 ± 10.7	80.2 ± 9.1
		Patients with genital warts HPV (−) *n* = 12	56.2 ± 33.8*	0.8 ± 1.8	177.1 ± 126.4	7.4 ± 0.3	56.2 ± 19.8*	36.3 ± 14.4	81.3 ± 10.5
		Subjects with HPV+ partner HPV (+) *n* = 27	48.5 ± 23.0*	2.8 ± 1.2	172.8 ± 110.2	7.6 ± 0.2	38.4 ± 13.2*	31.8 ± 11.2	82.4 ± 8.8
		Subjects with HPV+ partner HPV (−) *n* = 39	50.1 ± 22.3*	2.5 ± 1.3	178.4 ± 102.3	7.7 ± 0.4	53.8 ± 16.5*	31.8 ± 11.2	82.4 ± 8.8
		Infertile patients HPV (+) *n* = 11	30.0 ± 21.5*	2.9 ± 1.9	99.4 ± 88.8	7.7 ± 0.3	33.9 ± 15.9*	32.9 ± 13.9	79.8 ± 8.6
		Infertile patients HPV (−) *n* = 97	35.2 ± 23.0*	3.0 ± 1.5	102.9 ± 100.9	7.6 ± 0.3	51.7 ± 16.2*	33.1 ± 11.1	84.6 ± 10.7
		Fertile controls HPV (+) *n* = 2	60.5 ± 31.5*	2.5 ± 1.6	175.5 ± 131.6	7.6 ± 0.2	55.5 ± 17.6*	33.5 ± 10.6	81.7 ± 9.4
		Fertile controls HPV (−) *n* = 88	58.7 ± 30.8*	2.6 ± 1.6	176.0 ± 139.6	7.7 ± 0.2	54.2 ± 17.9*	33.0 ± 13.5	83.9 ± 8.0

Foresta et al. (2011b) [33]	Cross-sectional clinical	Infected infertile patients *n* = 32	32.4 ± 21.1	3.0 ± 1.1	100.2 ± 73.4	7.6 ± 0.3	29.7 ± 13.8*	17.8 ± 9.1	78.3 ± 11.6

Garolla et al. (2012) [17]	Case-control	HPV-infected patients *n* = 22	29.0 ± 10.3	3.1 ± 0.9	87.7 ± 36.3	7.6 ± 0.2	29.6 ± 14.2*	19.0 ± 6.3	81.3 + 6.3
		Control subject *n* = 13	30.5 ± 9.8	3.3 ± 1.0	98.8 ± 46.7	7.5 ± 0.3	42.4 ± 22.7*	21.1 ± 7.5	83.8 + 8.3
		L1-incubated sperm (pool)					22.6 ± 8.7*	20.9 ± 6.5	82.8 + 8.7

Garolla et al. (2013) [34]	Cross-sectional clinical	Infertile HPV-infected patients *n* = 61	32.0 ± 11.2^+^		94.2 ± 36.5^+^		29.0 ± 11.4^+^	18.8 ± 6.2	80.0 ± 7.1
		Infertile noninfected patients *n* = 104	34.6 ± 9.8^+^		108.8 ± 44.5^+^		47.8 ± 11.0^+^	18.5 ± 4.3	83.2 ± 5.1
		Control subjects *n* = 92	51.3 ± 8.4^+^		156.0 ± 42.9^+^		53.4 ± 11.4^+^	21.3 ± 4.7	83.6 ± 5.1

^+^ *P* < 0.01; *P < 0.05.

**Table 4:** Data about studies analyzing exogenous sperm HPV-DNA vehicle and its impact on product of conception.

Authors (year)	Number of samples	Aim of the study	Study setting	Main outcomes	Conclusions
Chan et al. (1994) [35]	42	Detect presence of HPV-DNA 16/18 in sperm cells	HPV-DNA detection trough PCR	HPV-DNA detection (PCR): HPV 16: 64.3% HPV 18: 38.1% HPV L1: 35.7%	(i) The study demonstrated the presence of HPV-DNA in sperm cells (ii) The result suggest a possible role of sperm as a vector for HPV

Lai et al. (1997) [36]	24	Detect presence of HPV-DNA 16/18 in both seminal plasma and sperm cells	HPV-DNA detection trough PCR	HPV-DNA detection (PCR): HPV 16: 33.3% (8.3% seminal plasma; 25% sperm cells) HPV 18: 79.1% (33.3% seminal plasma, 45.8% sperm cells)	(i) HPV can infect both sperm cell and seminal plasma (ii) HPV-DNA 16 and 18 are expressed actively in infected sperm cells (iii) The HPV infected sperm cells can behave as vectors for the transmission of HPV to fetuses through fertilized eggs

Chan et al. (1996) [37]	—	Demonstrate that sperm cells transfected with HPV-DNA 16/18 can vehicle viral genome to cells of uterus and embryo	(i) Non-human (mouse) experimental study (ii) HPV-DNA detection trough PCR	(i) Blastocyst took-up HPV-DNA fragments from both HPV-DNA 16 and HPV-DNA 18 of carrier sperm (ii) The sperm cells transferred HPV-DNA18 but not 16 fragments to the uterine cells at both the cervical-uterine end and the uterine-tubal end	(i) It is possible to apply the transmission of HPV DNA from the sperm to the embryos and cells of the reproductive tract (ii) The study suggests sperm as a vector for the transmission of DNA to the developing embryo

Cabrera et al. (1997)	—	Demonstrate that exogenous HPV-DNA taken into blastocysts is localized to both the inner cell mass and trophoblast cells	(i) Non-human (mouse) experimental study (ii) Mouse blastocysts exposed to migrating human sperm cells carrying exogenous DNA fragments from HPV 16 and 18 (iii) HPV-DNA detection trough PCR	Mouse blastocysts transfected by carrier sperm with HPV-DNA 16 and 18 showed localization of the HPV- DNA to both the inner cell mass and trophoblast cells	(i) Exogenous DNA taken into blastocysts is localized to both the inner cell mass and trophoblast cells (ii) Only live sperm exhibited the capacity to carry various sizes of exogenous DNA, suggesting the involvement of active cell membrane mechanism in the transference process

Lee et al. (2001)	8	Demonstrate that HPV-DNA can induce sperm cell apoptosis through the determination of p53 exons 5 and 8 integrity	Sperm apoptosis was detected trough Comet assay	Sperm apoptosis (Integrity of exons 5 and 8 of the p53 gene) Exon 5 ratio Exon 8 ratio HPV 16 1.35 ± 0.38 0.76 ± 0.03 HPV 18 0.86 ± 0.05 1.14 ± 0.26 HPV 6b/11 1.26 ± 0.21 1.06 ± 0.15 HPV 31 0.91 ± 0.20 0.91 ± 0.01 HPV 33 0.98 ± 0.06 1.19 ± 0.18	The data suggest that different HPV types preferentially degrade different exons of important genes
Connelly et al. (2001) [21]	6	Demonstrate that HPV-DNA can induce sperm cell apoptosis	Sperm apoptosis was detected trough DNA disc chip assay	Percentage of sperm apoptosis *(Sperm head density: pixels, mean ± SEM): Control (DQA1) 167.3 ± 2.4 HPV 16 125.9 ± 3.8 HPV 18 163.2 ± 3.3 HPV 31 154.0 ± 3.5 HPV 33 166.7 ± 3.7 HPV 6/11 168.0 ± 2.8	(i) HPV types 16 and 31 might lead to failed embryonic development through sperm apoptosis

You et al. (2002) [38]	—	(i) Determine if the DNA of trophoblast were disrupted by the presence of HPV DNA	(i) Use of recombinant adeno-associated viruses (rAAV) to introduce the HPV-16 E6 and E7 oncogenes into trophoblasts	HPV-16 oncogene expression may lead to outright trophoblast death	(i) These changes to trophoblast might be responsible of trophoblast and placental alteration and could contribute to spontaneous abortions

Calinisan et al. (2002) [39]	—	(i) Determine if the DNA of blastocysts was disrupted by the presence of HPV DNA (ii) Determine if the intensity of DNA damage was associated with the type of HPV	(i) Non-human (mouse) experimental study (ii) Blastocysts infected with HPV-DNA 16, 18, 31, or 33 (iii) Blastocyst apoptosis was detected trough Comet assay	Mouse blastocysts apoptosis (DNA fragmentation) (mean pixel intensity ± 1 SD)^§^ HPV 16: 190.1 ± 24.4^+^ HPV 18: 180.1 ± 21.7^+^ HPV 31: 171.9 ± 33.2^+^ HPV 33: 179.5 ± 12.0^+^	(i) Only HPV-DNA 16 was associated with significant DNA fragmentation (ii) The intensity of DNA damage was not linked to the specific type of HPV

Henneberg et al. (2006) [40]	—	(i) Assess the development of early embryos exposed to HPV DNA (ii) Analyze the blastocyst hatching process after HPV exposure.	(i) Non-human (mouse) experimental study (ii) Two-cell and 4–8-cell mouse embryos exposure to HPV-DNA 16 and 18	(i) HPV 16 and 18 inhibited two-cell embryo development but not 4–8-cell stage (ii) 25.9% less blastocyst formed with HPV 16 exposure (iii) 25.9–31.8% more degenerated embryos with HPV 16 exposure	(i) Demonstration of HPV embryo stage-specific effects on early development (ii) HPV 16 was shown to decrease blastocyst formation (iii) HPV 18 inhibited the blastocyst hatching process

Gomez et al. (2008) [41]	—	(i) Determine if HPV infection of extravillous trophoblast cells reduces cell invasion and induce apoptosis (ii) Determine if placental infection is associated with adverse reproductive outcomes attributed to placental dysfunction	(i) Extravillous trophoblast cells transfected with the HPV-16 genome were detected (ii) Apoptosis assay (Cell Death Detection ELISA) (iii) Invasion assays (Cell Invasion Assay Kit)	(i) Rates of apoptosis were 3- to 6- fold greater in transfected cells than in non-transfected cells^†^ (ii) Invasion of transfected cells through extracellular matrices was 25–58% lower than that of the controls^#^	(i) HPV extravillous trophoblast infection induces cell death and may reduce placental invasion into the uterine wall (ii) HPV infection may cause placental dysfunction and could be associated with adverse pregnancy outcomes, (such as preterm delivery

Foresta et al. (2011a) [25]	—	(i) HPV localization in sperm cell (ii) Demonstrate that HPV-DNA transfected sperm cells can transfer viral genome into oocytes	(i) Fluorescence in situ hybridization for HPV (FISH) (ii) Hamster Egg Penetration Test (HEPT) with human sperm transfected with HPV E6/E7 plasmid	(i) HPV is localized at the equatorial region of sperm head through interaction between the HPV capsid protein L1 and syndecan-1 (ii) HPV transfected sperms are able to penetrate the oocyte (iii) Viral genes are then activated and transcribed into the oocyte	(i) Sperm might function as vectors for HPV transfer into the oocytes

*Lower head intensity (in pixels) represented greater DNA fragmentation. (The data were expressed as mean ± SEM) ^§^Lower pixel intensity is associated with more DNA fragmentation.

^+^ *P* < 0.05; ^†^ *P* < 0.01; ^#^ *P* < 0.001.
